# Evaluating the Sealing Capacities of Different Endotracheal Tube Cuff Designs

**DOI:** 10.1089/respcare.12465

**Published:** 2025-08-04

**Authors:** Junichi Michikoshi, Makoto Yamamoto, Kumiko Takagi, Takeko Kudo, Yoshihiro Tange, Tadashi Tomo

**Affiliations:** ^1^ Drs. Michikoshi, Tange, and Tomo are affiliated with Department of Advanced Medical Sciences, Faculty of Medicine, Oita University, Yufu City, Oita, Japan.; ^2^ Dr. Yamamoto, Ms. Takagi, and Ms. Kudo are affiliated with Oita Kyowa Hospital, Oita City, Oita, Japan.

**Keywords:** mechanical ventilation, artificial respiration, endotracheal intubation, cuff, ventilation, ventilator-associated pneumonia

## Abstract

**Background:**

Endotracheal tube (ETT) cuffs prevent over-the-cuff secretions from flowing into the lower airways. However, they may not completely prevent fluid leakage around ETTs. To validate a cuff design with high sealing capacity, we compared ETTs with varying cuff materials and structural properties.

**Methods:**

We used 7 ETTs with different cuff materials (polyvinyl chloride [PVC], polyurethane [PU]), shapes (conical, tapered, or cylindrical), wall thickness, and cuff sizes (contact area with the trachea). Wall thickness was measured after cutting the cuff using a micrometer. The contact area was calculated based on the long diameter of the cuff when expanded within a clear acrylic tube. The sealing capacity was defined as the time taken for 10 mL of distilled water to leak past the cuff. The sealing capacities of the cuffs were compared by inserting them into the simulated trachea (silicone corrugated tube).

**Results:**

The wall thicknesses were 29–29.3 µm for PU and 45.6–285 µm for PVC cuffs. The contact areas with the trachea were 18.7–27.1 and 12.5–22.3 cm for PU and PVC cuffs, respectively. The mean (SD) sealing capacities were 2,381 (484), 437 (177), 56 (12), and 24 (4) s for the cylindrical PU, conical PU, tapered PVC, and conical PVC cuffs, respectively. For the 3 cylindrical PVC cuffs (A, B, and C, respectively), the mean (SD) sealing capacities were 53 (11), 15 (2), and 9 (2) s.

**Conclusions:**

The PU cuff had a higher sealing capacity than the PVC cuff, and the cylindrical cuff had a higher sealing capacity than the conical cuff. For the PVC cuff, thinner materials had higher sealing capacities. Furthermore, the contact area between the cuff and model trachea significantly affected the sealing capacity.

## Introduction

Endotracheal tubes (ETTs) are important therapeutic devices used for the ventilatory management of patients with severe respiratory failure. They are also used for mechanical ventilation during surgical procedures performed under general anesthesia. All ETTs have an attached cuff, the role of which is to create a seal within the airway, allowing air flow through the ETT while preventing secretions from leaking past the cuff. To provide an adequate seal and prevent complications, it is recommended that cuff pressure be maintained at 20–30 cm H_2_O.^1,2^

Mechanically ventilated patients can develop severe pneumonia if any secretions from above the ETT cuff bypass the cuff toward the lungs. Ventilator-associated pneumonia (VAP) is an important variable that affects the prognosis of patients with severe pneumonia.^3,4^ As a proper seal between the trachea and ETT is crucial to preventing VAP, ETT cuff performance is likely a key factor influencing its successful prevention.^5^

Various companies have marketed ETTs with cuffs that vary in shape, material, dimension, and wall thickness. These features may influence cuff pressure maintenance and sealing capacity. However, no prior studies have compared the sealing capacity of ETT cuffs based on shape, wall thickness, and size.^6–9^ Furthermore, previous studies have utilized vertical, smooth-walled acrylic or silicone tubes with smooth surfaces as mock tracheas, but these do not fully resemble a real trachea. Therefore, we aimed to compare the sealing capacities of various ETT tubes with varying shapes, sizes, and wall thicknesses using a corrugated silicone tube as a model trachea.

**QUICK LOOK**

**Current knowledge**

Endotracheal tracheal tube (ETT) cuff sealing capacity plays an important role in preventing ventilator-associated pneumonia (VAP). Several studies have evaluated the sealing capacity of cuffs and confirmed that polyurethane (PU) cuffs have high sealing capacities. However, these studies utilized smoothed-walled model tracheas, which do not accurately replicate the shape of a real one. Additionally, the influence of cuff material, wall thickness, shape, and size (area of contact with the trachea) on the sealing capacity has not been comprehensively explored.

**What this paper contributes to our knowledge**

The overall cuff sealing capacities of 7 different cuff designs were compared using a semi-realistic corrugated silicone tube as a model trachea. The cylindrical PU cuff demonstrated the best sealing capacity, which could be attributed to its large area of contact with the model trachea. Regarding polyvinyl chloride cuffs, the cuff wall material thickness, shape, and size (area of contact with the trachea) significantly influenced the sealing capacity. ETT cuff designs prioritizing thin cuff walls and large areas of contact with the trachea have improved sealing capacity.

## Methods

We evaluated 7 types of tracheal tubes (inner diameter: 8.0 mm) with different materials and structural designs: MICROCUFF (Avanos Medical, Alpharetta, GA, USA; cylindrical polyurethane [PU] cuff), Seal Guard Evac (Covidien, Dublin, Ireland; conical PU cuff), Taper Guard Evac (Covidien; tapered polyvinyl chloride [PVC] cuff), SACETT (Portex Blue Line SACETT; Smiths Medical, Weston, MA, USA; conical PVC cuff), Hi-Lo Evac (Covidien; cylindrical PVC cuff [A]), Parker Flex-Tip (Parker Medical, Highlands Ranch, CO, USA; cylindrical PVC cuff [B]), and Trafit (SENKO MEDICAL INSTRUMENT, Tokyo, Japan; cylindrical PVC cuff [C]) (Supplementary Figure S1). Five of each type of ETTs were used in the study.

The horizontal length and wall thickness of the cuffs were measured using a Digimatic micrometer (MDC-25MX and MDE-50M; Mitutoyo, Kanagawa, Japan). The horizontal lengths were measured after setting the cuff pressure to 25 cm H_2_O. Cuff wall thickness was measured at the vertical center of the cuff after cutting it open. Each cuff was measured 10 times.

To measure the contact area between the cuff and the model trachea, each cuff was inserted into a clear acrylic tube (inner diameter: 1.8, 2.0, 2.2, and 2.4 cm) and inflated to a pressure of 25 cm H_2_O. A tape measure was used to measure 4 vertical lines of contact between the cuff surface and the trachea at 4 circumferential points at 90° apart. The contact surface area was calculated from the average of the 4 lengths using the equation presented in Supplementary Figure S2. Each acrylic tube was measured 15 times.

The sealing capacity was measured by inserting the ETT into a model trachea (silicone corrugated tube; inner diameter, 17–25 mm; Puritan Bennett Tube Assy silicone 21″; Covidien), and a continuity cuff pressure controller (Cuff Keeper, TOKSO, Oita, Japan) was used to maintain a cuff pressure of 25 cm H_2_O.^10^ Distilled water was injected into the upper cuff compartment; leakage of the water into the lower compartment was quantified using an electronic balance (FZ-300iWPR; A&D, Tokyo, Japan). The cuff sealing capacity was defined as the time (s) required for 10 mL of distilled water to leak into the lower compartment (Supplementary Figure S3). A longer inflow time indicated a higher sealing capacity. Each model trachea was measured 19 times.

### Statistical analyses

All statistical analyses were performed using R version 3.32 (R Foundation for Statistical Computing, Vienna, Austria) and EZR (version 1.36; Jichi Medical University, Saitama Medical Center, Saitama, Japan) software. Data were analyzed using one-way analysis of variance and the Friedman test with Bonferroni correction. Statistical significance was set at *P* < .001. Correlations were evaluated using Pearson correlation coefficient (*r*). Data are reported as the mean (SD) unless otherwise specified.

## Results

The cuff shape, water inflow time and volume, horizontal cuff length and wall thickness, vertical length of the cuff in each model trachea, and contact area between the cuff and each model trachea are presented in Table 1.

**Table 1.** Specifications of the tested cuffs

Material	PU	PU	PVC	PVC	PVC	PVC	PVC
***Shape***	***Cylindrical***	***Conical***	***Tapered***	***Conical***	***Cylindrical (A)***	***Cylindrical (B)***	***Cylindrical (C)***
Inflow time (s/10 mL)	2381 (484)	434 (177)	56 (12)	24 (4)	53 (11)	15 (2)	9 (2)
Horizontal length (mm)	28.5 (0.2)	28.8 (0.2)	26.7 (0.3)	28.9 (0.4)	33.4 (0.4)	24.2 (0.1)	25.7 (0.3)
Wall thickness (µm)	29.0 (9.0)	29.3 (7.7)	72.7 (12.4)	81.5 (12.4)	45.6 (9.5)	113 (10.7)	285 (20.0)
***Model trachea diameter***	***Vertical length of cuff in each model tracheal diameter (cm)***						
1.8 cm	4.34 (0.11)	3.72 (0.11)	2.61 (0.18)	3.05 (0.07)	3.52 (0.18)	3.12 (0.05)	3.23 (0.10)
2.0 cm	4.25 (0.10)	3.31 (0.09)	2.26 (0.15)	2.81 (0.12)	3.51 (0.11)	3.01 (0.06)	2.95 (0.05)
2.2 cm	4.12 (0.13)	2.67 (0.17)	1.72 (0.09)	2.43 (0.11)	3.32 (0.15)	2.67 (0.08)	2.68 (0.07)
2.4 cm	3.81 (0.16)	1.92 (0.13)	1.21 (0.11)	2.03 (0.21)	3.23 (0.06)	2.36 (0.08)	2.32 (0.09)
Mean (SD)	41.3 (2.0)	29.1 (6.8)	19.5 (5.3)	25.9 (3.9)	34.0 (1.2)	27.9 (3.0)	28.0 (3.4)
***Model trachea diameter***	***Contact area between cuff and model trachea in each tracheal diameter (cm^2^)***						
1.8 cm	24.6 (0.67)	21.1 (0.64)	14.8 (1.03)	17.3 (0.41)	19.9 (1.06)	17.7 (0.32)	18.3 (0.62)
2.0 cm	26.7 (0.63)	20.8 (0.57)	14.2 (0.96)	17.7 (0.76)	22.1 (0.72)	18.9 (0.40)	18.6 (0.31)
2.2 cm	28.5 (0.95)	18.5 (1.20)	11.9 (0.64)	16.8 (0.79)	23.0 (1.04)	18.5 (0.61)	18.6 (0.55)
2.4 cm	28.7 (1.27)	14.5 (1.03)	9.14 (0.88)	15.3 (1.64)	24.4 (0.46)	17.8 (0.66)	17.5 (0.71)
Mean (SD)	27.1 (1.7)	18.7 (2.6)	12.5 (2.2)	16.8 (0.9)	22.3 (1.6)	18.2 (0.5)	18.2 (0.4)

Values are presented as means (SD).

PU, polyurethane; PVC, polyvinyl chloride.

**Fig. 1.**
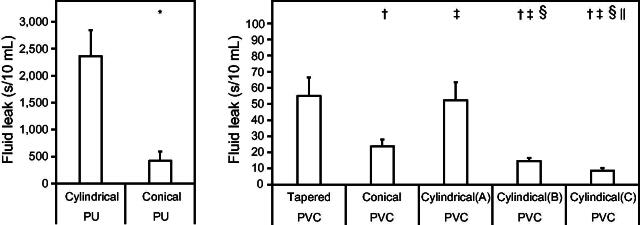
Results of the water leak test for each cuff design. *P < .01 versus cylindrical PU cuff. ^†^*P* < .01 versus tapered PVC cuff. ^‡^*P* < .01 versus conical PVC cuff. ^§^*P* < .01 versus cylindrical PVC cuff (A). ^||^*P* < .01 versus cylindrical PVC cuff (B). PU, polyurethane; PVC, polyvinyl chloride.

PU cuffs had significantly less leakage than PVC cuffs. The cylindrical PU cuff had significantly less leakage than the conical PU cuff (2,381 [484] vs 434 [177] s/10 mL, *P* < .001). Among the PVC cuffs, the leakage of the tapered cuff (56 [12] s/10 mL) was significantly less than that of the other shapes, except for the cylindrical PVC cuff (A: 53 [11] s/10 mL). Other cylindrical PVC types (B: 15 [2] s/10 mL; C: 9 [2] s/10 mL) had significantly increased leakage volumes (*P* < .001) (Fig. 1).

**Fig. 2.**
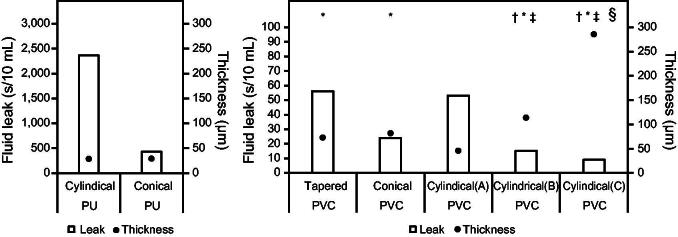
Sealing capacity of each cuff material. ^*^P < .01 versus cylindrical PVC cuff (A). ^†^*P* < .01 versus tapered PVC cuff. ^‡^*P* < .01 versus conical PVC cuff. ^§^*P* < .01 versus cylindrical PVC cuff (B). PU, polyurethane; PVC, polyvinyl chloride.

Figure 2 demonstrates the relationship between the cuff wall thickness and sealing capacity. Regarding the PU cuffs, no correlation was observed between the wall thickness and sealing capacity. However, thinner cuff materials resulted in a greater sealing capacity for PVC cuffs (*r* = .891, 95% CI [CI], 0.835–0.928).

The area of contact between the cuff and the model trachea varied with the tracheal diameter. The cuff shape and horizontal length were the major factors that affected the contact area.

**Fig. 3.**
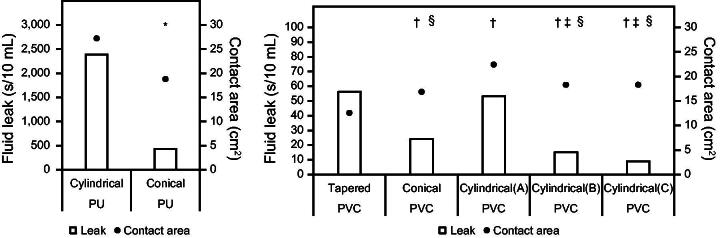
Relationship between the average area of contact between each cuff and the model trachea and the sealing capacity. ^*^P < .01 versus contact area between the cylindrical PU cuff and the model trachea. ^†^*P* < .01 versus contact area between the tapered PVC cuff and the model trachea. ^‡^*P* < .01 versus contact area between the conical PVC cuff and the model trachea. ^§^*P* < .01 versus contact area between the cylindrical PVC cuff (A) and the model trachea. PU, polyurethane; PVC, polyvinyl chloride.

Figure 3 illustrates the sealing capacities of the 7 cuff types and their relationships with the average area of contact with the model trachea. The cylindrical PU cuff (27.1 [1.7] cm^2^) had a significantly larger contact area and higher sealing capacity than the conical PU cuff (18.7 [2.6] cm^2^) (*P* < .001). The tapered PVC cuff (12.5 [2.2] cm^2^) had a significantly higher sealing capacity than the other PVC cuffs (16.8 [0.9]–22.3 [1.6] cm^2^) (*P* < .001), despite having the lowest contact area. Regarding the cylindrical PVC cuffs, a larger contact area resulted in a greater sealing capacity.

## Discussion

There are many ETTs currently available for clinical use. VAP prevention is dependent on minimizing the flow of secretions past the cuff into the lungs. However, various types of ETT cuffs have different sealing capacities. Earlier experiments evaluating the sealing capacity of ETT cuffs used a straight-walled tracheal model made of acrylic or silicone material with a smooth surface.^6–9^ This may have influenced the evaluation of the cuff sealing capacity. Therefore, we conducted a comprehensive comparative study using different ETTs with cuffs of varying materials, wall thickness, shape, and size in a simulated trachea that closely resembles a real one. The results showed that cuffs made of PU, which are thinner-walled and have a larger contact area with the trachea, boast a higher sealing capacity against secretion leakage.

Our results showed a significantly higher sealing capacity of the PU than of the PVC cuffs. A substantial difference in sealing capacity was observed between the 2 materials regardless of the cuff design. The influence of shape on sealing capacity became apparent when PU and PVC cuffs were compared; cylindrical designs were favorable for PU cuffs, whereas PVC cuffs had the highest sealing capacity with a tapered shape. The PVC cuffs also demonstrated a wider range of sealing capacities with the cylindrical design.

Furthermore, we examined whether the difference in the sealing ability was attributable to the cuff wall material thickness. As shown in Figure 2, less leakage was observed with thinner-walled PVC cuffs, suggesting that the thickness of the cuff wall material affects the sealing ability.

We focused on the area of contact between the cuff and the trachea to examine the influence of the cuff shape on its sealing ability. As shown in Figure 3, the contact area varied with the diameter of the trachea, suggesting that the horizontal cuff length relative to the diameter of the trachea is related to the sealing ability of the cuff. Furthermore, higher sealing capacities were observed with wider contact areas for PU cuffs; for PVC cuffs, this relationship was observed only in the cylindrical type.

In the present study, a silicone corrugated tube was used as the model trachea; we believe this may have revealed more pronounced differences in the sealing properties of ETT cuff materials. In a previous study, Rozycki et al employed a novel biorealistic animal-like tracheal model that was comparable with that of an adult human trachea to assess leakage from cylindrical PU and cylindrical and tapered PVC cuffs using mucus simulants. Similar to our findings, they found variations in leakage across the different cuff materials and shapes.^11^

PU cuffs have been reported to reduce microaspiration and the frequency of VAP in clinical practice,^12–16^ with cylindrical variants being substantially more effective than conical variants. This was evident not only because of the thin material but also because of the larger contact area with the tracheal surface, resulting in a higher sealing capacity. It was also thought that the thinner material conforms more readily to uneven surfaces and allows the material to adhere more closely to the inner tracheal surface, which enhances the sealing capacity of the cuff.

Conversely, PVC cuffs had lower sealing capacities, which was likely because of their thicker walls. On considering the shapes of both conical and cylindrical cuffs, thicker cuff wall materials were associated with an increased fluid flow past the cuff. This is likely attributable to the cuff size. The coronal and sagittal diameters of the tracheal air column, respectively, are reported to be 13–25 and 13–27 cm for men and 10–21 and 10–23 cm for women.^17^ When a cuff larger than the tracheal diameter is inflated, the cuff material is pleated excessively and forms folds, which has been associated with the leakage of secretions past the cuff.^18,19^

Therefore, the higher sealing capacity of thinner-walled cuffs is likely attributed to their small leakage area owing to fold formation; thicker-walled cuffs tend to have a larger fold-formation leakage area, and this reflects their inferior sealing capacity. Based on the above results, we believe that the lateral diameter is contributes to the thickness of the cuff material and the degree of folding, which is one of the causes of microaspiration. This indicates that the cuff size influences its sealing capacity, warranting further investigation. We posit that ETTs with improved cuff sealing capacities will be developed in the future based on these results.

This study had several limitations. First, it evaluated the sealing capacity using distilled water, which does not resemble the consistency of tracheal mucus secretions. Second, as the experiments were conducted under artificial conditions, the results may differ from those obtained in actual clinical settings. Third, the effects of PEEP were not taken into account because the model was not ventilated. Fourth, this study did not demonstrate a relationship with clinical VAP. Fifth, the pressure applied to the trachea and the pharyngeal injury were not analyzed. Finally, we did not evaluate the occurrence of cuff folds based on differences in the material thickness of the cuff wall and cuff size relative to the tracheal diameter. To confirm these results, further studies addressing these limitations are required.

## Conclusions

PU cuffs, particularly those that were cylindrical and had the largest contact area with the model trachea, demonstrated the highest sealing capacity against fluid leakage. The sealing capacities of PVC cuffs increased as the cuff wall thickness decreased, and the tapering of the cuff and its contact area with the model trachea had significantly affected the sealing capacity. ETT cuff designs prioritizing thin cuff walls and a large area of contact with the trachea may contribute to VAP prevention by improving sealing capacity.
