# Supplementary material for: Evaluating the Sealing Capacities of Different Endotracheal Tube Cuff Designs
Source: Respir Care. 2025 Aug 4;70(8):962–7. doi: 10.1089/respcare.12465 (PMC12411406; doi:10.1089/respcare.12465)
Supplement: Supplementary Figure S1 [file respcare.12465_supplementary_figures1.pptx]

## Slide 1
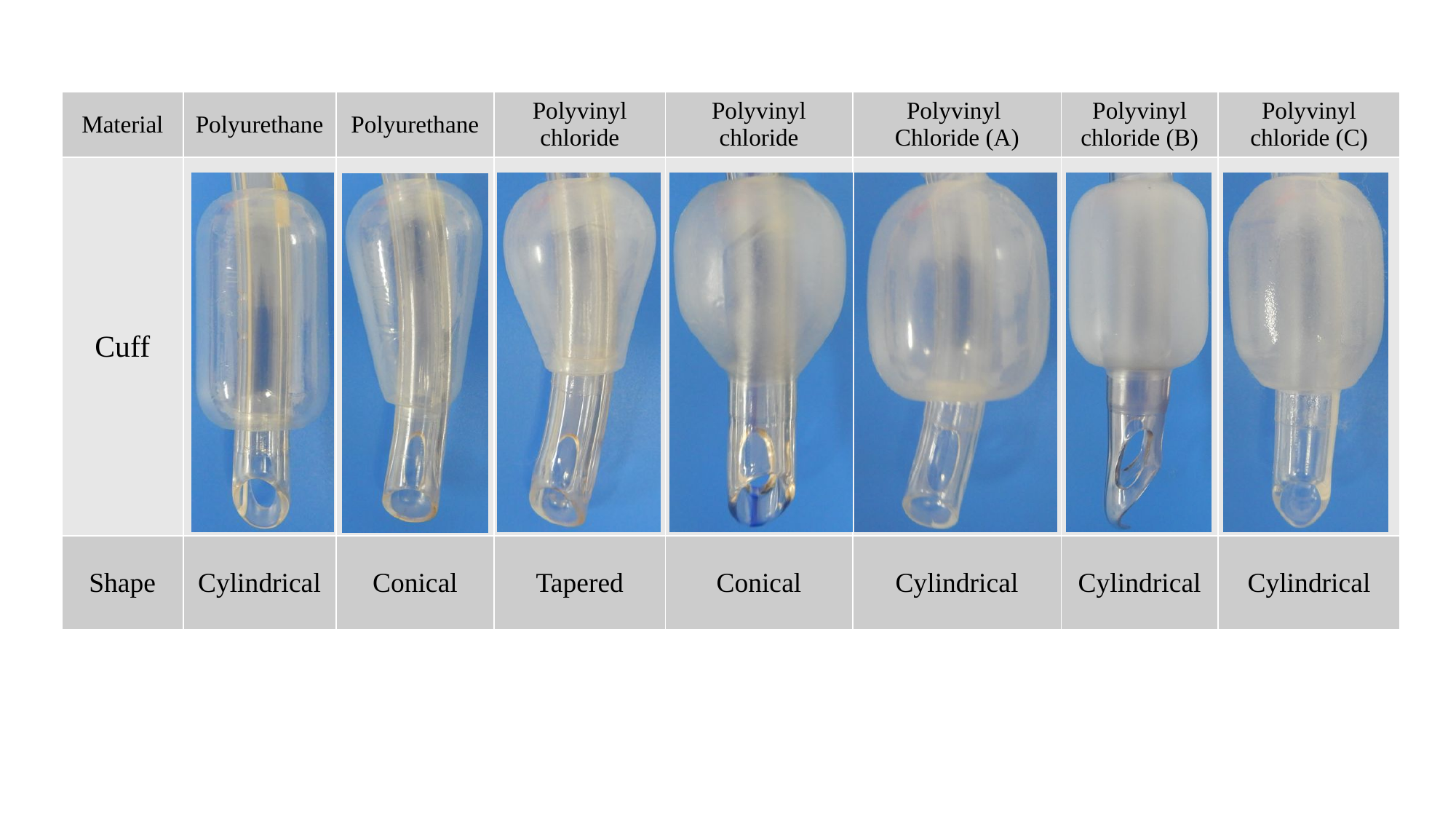

| Material | Polyurethane | Polyurethane | Polyvinyl chloride | Polyvinyl chloride | Polyvinyl Chloride (A) | Polyvinyl chloride (B) | Polyvinyl chloride (C) |
| --- | --- | --- | --- | --- | --- | --- | --- |
| Cuff | | | | | | | |
| Shape | Cylindrical | Conical | Tapered | Conical | Cylindrical | Cylindrical | Cylindrical |
