# Supplementary material for: Evaluating the Sealing Capacities of Different Endotracheal Tube Cuff Designs
Source: Respir Care. 2025 Aug 4;70(8):962–7. doi: 10.1089/respcare.12465 (PMC12411406; doi:10.1089/respcare.12465)
Supplement: Supplementary Figure S2 [file respcare.12465_supplementary_figures2.pptx]

## Slide 1
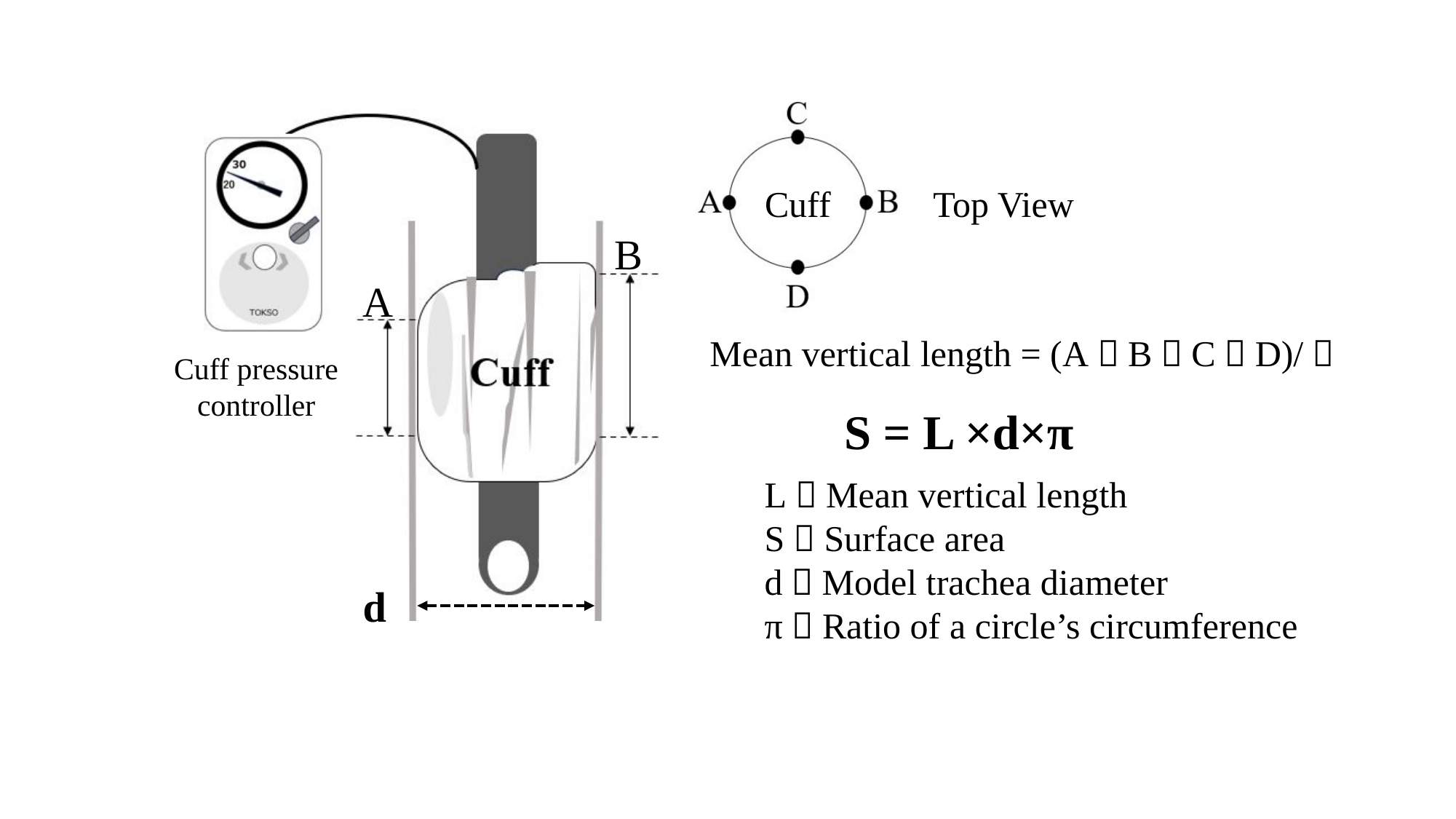

Cuff
Top View
B
A
Mean vertical length = (A＋B＋C＋D)/４
Cuff pressure
controller
S = L ×d×π
L：Mean vertical length
S：Surface area
d：Model trachea diameter
π：Ratio of a circle’s circumference
d
