# Supplementary figures and images for: Evaluating the Sealing Capacities of Different Endotracheal Tube Cuff Designs
Source: Respir Care. 2025 Aug 4;70(8):962–7. doi: 10.1089/respcare.12465 (PMC12411406; doi:10.1089/respcare.12465)

## Slide 1
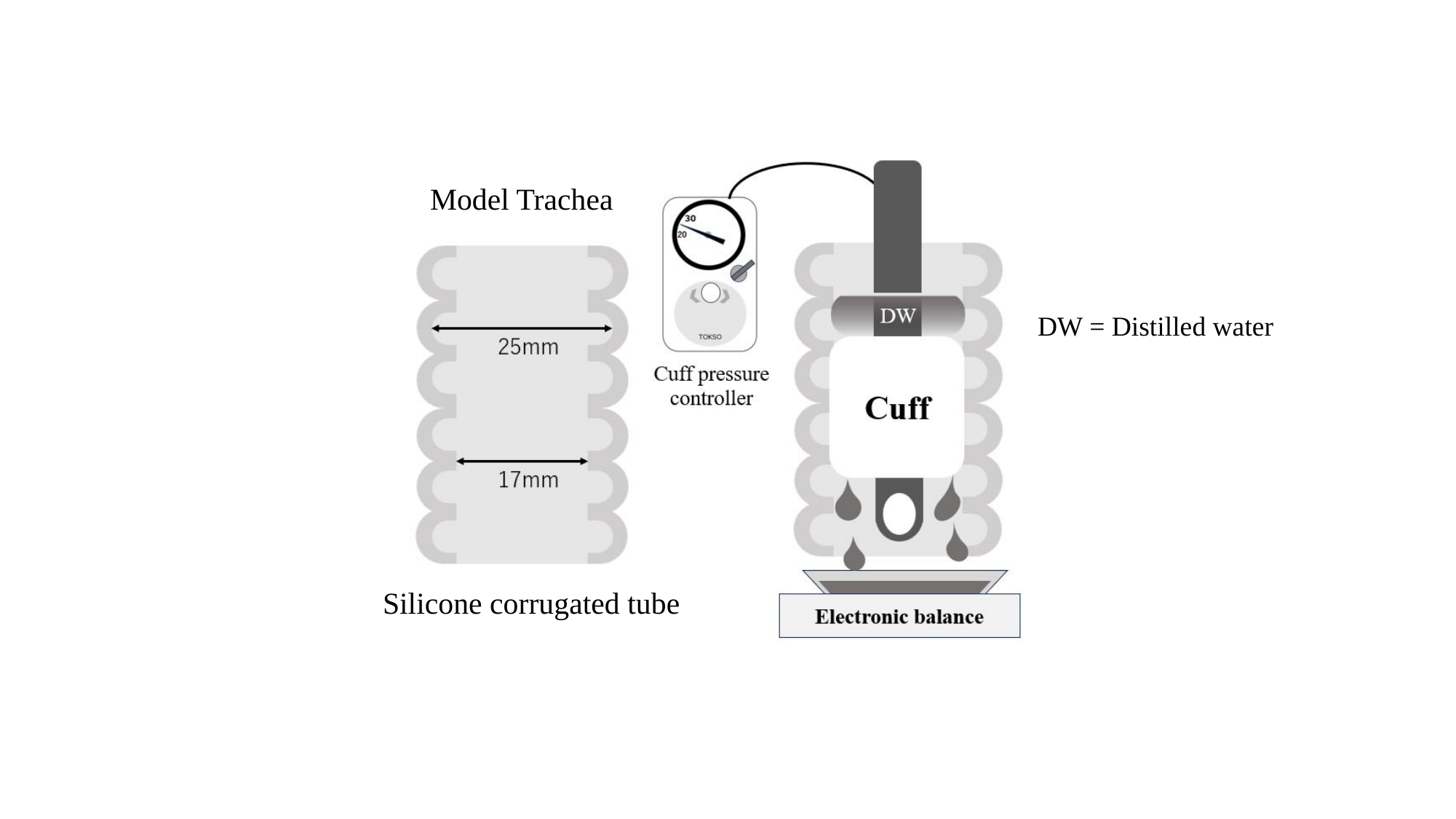

Model Trachea
DW = Distilled water
Silicone corrugated tube

Supplement: Supplementary Figure S3 [file respcare.12465_supplementary_figures3.pptx]
